# Rift Valley Fever outbreaks in the East African Community: insights from ProMed data (2010–2024)

**DOI:** 10.3389/fpubh.2024.1298594

**Published:** 2024-12-11

**Authors:** Pacifique Ndishimye, Therese Umuhoza, Brigitte Umutoni, Fathiah Zakham, Matin Ndayambaje, Benjamin Hewins, Methode Ngabo Gasana, Ali Toloue Ostadgavahi, Gustavo Sganzerla, Fabrice Ndayisenga, David Kelvin, Jean Claude Udahemuka

**Affiliations:** ^1^Epidemic Response Laboratory, Research and Innovation Centre, African Institute for Mathematical Sciences (AIMS), Kigali, Rwanda; ^2^Stansile Research Organization, Kigali, Rwanda; ^3^Stansile Research Organization, Halifax, NS, Canada; ^4^Program for Monitoring Emerging Diseases (ProMed), Brookline, MA, United States; ^5^Department of Microbiology and Immunology, Dalhousie University, Halifax, NS, Canada; ^6^Rwanda Agriculture and Animal Resources Development Board, Kigali, Rwanda; ^7^Department of Veterinary Medicine, University of Rwanda, Nyagatare, Rwanda

**Keywords:** Rift Valley Fever, outbreak, ProMed, mosquito-borne disease, East African Community

## Abstract

**Background:**

Rift Valley Fever (RVF) is a mosquito-borne zoonotic disease that poses a serious threat to both humans and livestock across various regions, particularly in Africa, the Arabian Peninsula, and parts of the Indian Ocean Islands. This study seeks to analyze the spatial and temporal distribution and trends of RVF outbreaks within the East African Community (EAC) countries, offering insights into the patterns and progression of these outbreaks in the region.

**Methods:**

We conducted a retrospective analysis of the Program for Monitoring Emerging Diseases (ProMed), a digital, event-based disease surveillance system, to identify reports of outbreak events in Uganda, Kenya, Rwanda, Burundi, Tanzania, and South Sudan from 2010 to 2024. Outbreak events were systematically tabulated by year, and each record was reviewed to assess RVF outbreak characteristics, locations, trends, and spatial-temporal distribution over the past 14 years.

**Results:**

Between 2010 and 2024, 67 RVF outbreaks were documented across Uganda, Rwanda, Kenya, Tanzania, Burundi, and South Sudan, impacting both animal and human populations with confirmed cases and fatalities. Key interventions to contain these outbreaks included restricting animal movement, vaccination campaigns, disease awareness initiatives, enhanced surveillance, contact tracing, isolation, and treatment. Reporting of these outbreaks varied across regions, with a notable monthly increase in cases during May and June and the highest annual incidence observed in 2018.

**Conclusion:**

The recurrent and widespread outbreaks of Rift Valley Fever across East Africa highlight an urgent need for increased investment in research, surveillance, prevention, and control efforts to combat this disease.

## Introduction

Rift Valley Fever (RVF) virus is a mosquito-borne zoonotic arbovirus that causes disease in livestock and humans (1). RVFV is harbored and transmitted by multiple genera of mosquitos, such as the *Aedes, Anopheles, Culex* and *Mansonia* and may also be spread to humans via ingestion or contact with contaminated blood, bodily fluids, or tissues of infected animals (2). The RVF manifestation in livestock and ruminants is primarily acute and generally more severe for young and juvenile animals, where sheep and goats are the most susceptible animals to developing severe disease caused by the virus. Clinical symptoms for infected animals include hemorrhagic fever, lymphadenitis, nasal, and ocular secretions, vomiting, high abortion frequency, and a high rate of mortality in young animals (3, 4). The RVF in humans usually result in a self-limiting febrile illness, where roughly 50% of infected individuals report no clinical symptoms; importantly, however, a small percentage of human cases progress to severe disease, causing hemorrhagic fever, hepatic disease/failure, encephalitis, and death (3, 5).

A global risk map for RVF has highlighted potential transmission zones in Africa and the Middle East. This assessment considers geographic factors and a suitability indicator based on the Aedes mosquito, one of the RVF virus vectors, and the presence of susceptible hosts (6–8).

Furthermore, limited numbers of production animals in the arabian peninsula region leads also some countries like Yemen to import animals from the African horn. Recent reports have shown that some of the imported animals have undergone veterinary control screens whereas more than million animals are annually illegally imported via sea without any health control measures (9, 10). Although RVF primarily affects livestock, it also significantly threatens the stability of large-scale agricultural economies (11). Factors such as the expansion of the *Aedes* mosquito's range into Europe and the Americas, climate change and extreme weather events, and global travel could facilitate the spread of RVF to new regions, increasing the risk of infections in both animals and humans. The risk of an RVF outbreak is governed by a number of factors, including environmental conditions. Heavy rainfall periods have been classified as important risk factors for outbreaks as they create a suitable environment for mosquito proliferation (12). Moreover, linked global-trade chains have contributed by introducing infected livestock into non-endemic regions (13). Other economic factors such as informal and unregulated herding and the lack of sanitary mandates in abattoirs also facilitate infection (14). Since its causative agent, Rift Valley fever phlebovirus, was first isolated in 1930 in the Rift Valley region in Kenya, several major outbreak events have been reported in Africa and the Arabian Peninsula (15–17). Adequately controlling RVF spread relies on a variety of methods, such as monitoring, surveillance, and vaccination. Many of these methods are cost prohibitive and should therefore be approached using transdisciplinary solutions for disease management.

In Sub-Saharan Africa, specifically in East Africa, RVF outbreaks remain a significant public health concern (18, 19), especially in regions with high livestock populations where the virus is endemic. Recent reports indicate an upsurge in cases, largely attributed to seasonal rain patterns that create favorable breeding conditions for the Aedes and Culex mosquitoes responsible for transmitting the virus. Kenya, Uganda, and Tanzania have each seen periodic outbreaks, with concerns growing around cross-border transmission due to the high mobility of livestock and people in the region. Health officials are emphasizing the importance of ongoing surveillance, rapid response capabilities, and community awareness to mitigate the spread, alongside research into RVF immunogenicity and vaccine development to protect at-risk populations.

In East African countries (i.e., Burundi, Kenya, Rwanda, South Sudan, Tanzania, and Uganda), an unexpected RVF outbreak occurred in 2018 (20). This recent outbreak was unexpected as RVF outbreak events typically occur during El Niño periods with excessive rainfall. However, in 2018, there was an unusual deluge following a prolonged drought. Despite initial skepticism due to the late timing of the flooding, RVF outbreak events were reported in Kenya, Rwanda, and Uganda, with significant rainfall creating conditions conducive for the propagation of RVFV mosquito vectors. Risk maps indicated a broader area of potential activity, highlighting the unpredictability of climate-related disease outbreaks (8).

Outbreak events, case numbers, and human deaths caused by infection with RVFV have been exacerbated by the COVID-19 pandemic in East Africa (21, 22). Therefore, this review seeks to describe RVF outbreak events distribution and trends in the six East African countries over the past 14 years. Generated evidence will serve as a foundation for modeling future epidemiological studies, as well as providing baseline information to enhance mitigation strategies.

## Methods

### Data source

We conducted a retrospective analysis of the Program for Monitoring Emerging Diseases (ProMed), a digital disease monitoring system hosted by the International Society for Infectious Diseases (ISID) (23, 24), to identify reports of RVF outbreak events from the East Africa Community Partner States i.e., Uganda, Kenya, Rwanda, Burundi, Tanzania, and South Sudan. The ProMed database holds a record of major global outbreaks of emerging and re-emerging infectious diseases named the “ProMed mail database.” The ProMed database is also subdivided into regional official language specific databases, which included ProMed Anglophone Africa for English speaking countries (ProMed-EAFR), and Francophone Africa for French speaking countries (ProMed-FRA). Despite instances of overlap between posts recorded in ProMed-EAFR and ProMed-FRA for some EAC countries, both databases served as data sources for this review. We considered RVF outbreak events documented both in English and French in the period of 2010–2024. All RVF outbreak reports with suspected or requesting additional information were excluded. Specifically, reports were excluded if they lacked essential epidemiological details or if key data elements (such as case counts, location, or timeline) were missing or inconsistent, thus requiring additional verification. Our aim was to ensure that only fully verified and reliable reports were included in our analysis to maintain data integrity.

### Search strategy

To conduct searches in the ProMed database, we first defined relevant keywords and synonyms. This included the disease name (Rift Valley Fever or RVF or Fièvre de la vallée du Rift or FVR), associated syndromes (e.g., hemorrhagic fever), and countries within the East African Community (Rwanda, Uganda, Kenya, Tanzania, Burundi, and South Sudan). The search parameters covered a specified timeframe from January 2010 to July 2024 and allowed for both English and French language searches. Searches utilized logical operators “AND/OR” to refine results.

The initial search was conducted in the ProMed-EAFR database, enabling a targeted search strategy reviewed by two parallel reviewers. Any discrepancies in inter-rater assessments were resolved by a third-party reviewer. Additional searches were completed in ProMed-FRA and ProMedmail, with reference numbers from each post recorded in Microsoft Excel. As ProMed is an open-access database, ethical approval was not required for this review.

### Data extraction and management

The reviewers developed an Excel template including the list of variables of interest for data collection. The list of variables compiled from the ProMed databases contained the outbreak ID, day, month, and year of outbreak occurrence, geographical location (district/province/county, and country), population (Human, animal, or both), mortality status (Yes/No), and major interventions (awareness, contact tracing, isolation, movement restriction, surveillance, treatment, and vaccination). All extracted data were cross validated by two independent reviewers. An additional dataset of spatial coordinates (latitude/longitude) were collected from geocoded locations via Earth Pro (7.3 Google LLC) to ascertain the specific geographical locations of each outbreak. The African regional administrative boundaries, water bodies, and rivers were obtained in a shapefiles format from the Africa Open Access library. The collected shapefiles data were managed in qGIS (V 3.10.3-A Coruña) software (25).

### Data analysis and visualization

The dataset gathered from the ProMed database was reviewed, structured and assessed for errors. A descriptive analysis allowed us to observe the characteristics of each outbreak event, including a trend- and spatial distribution analysis. The number of RVF outbreak events that occurred were recorded per month, year, population, and geographic location. RVF outbreak events that reported mortality were counted per country. Outbreak events that had major interventions, such as movement control or vaccination, were recorded per country. These analyses were performed using a pivot table in Microsoft Excel. A description of the geographical distribution of RVF outbreak events was performed by visualizing outbreak occurrence per geographical location point. This visualization was completed by comparing the spatial coordinate data (latitude/longitude) with the number of outbreak events per country in qGIS. Following this, a trend analysis of RVF outbreak events were observed by monthly and yearly line plotting.

## Results

In total, 65 RVF outbreak events were reported in EAC countries (Table 1). Uganda and Rwanda each reported 17 RVF outbreak events. Burundi had 14 RVF outbreak events, followed by Kenya, which had 12 RVF outbreak events. Tanzania and South Sudan, on the other hand, reported 3 and 2 RVF outbreak events, respectively. Among the RVF outbreak events reported, 43 were in animals, 17 in Humans, and three in both populations. The mortality rate in animals were reported in 26 RVF outbreak events, whereas a human fatality rate was reported in 15 RVF outbreak events. Various major interventions were reported; these included movement control during 17 RVF outbreak events, animal vaccination campaigns during 13 RVF outbreak events, disease awareness during five RVF outbreak events, active surveillance during three RVF outbreak events, and contact tracing, supportive therapy/care, and isolation were reported during one RVF outbreak event.

**Table 1:** Characteristics of RVF outbreak events reported in EAC partner states through the Program for Monitoring Emerging Diseases (ProMed), 2010–2024.

**Variable/country**	**Description**	**Burundi**	**Kenya**	**Rwanda**	**South Sudan**	**Tanzania**	**Uganda**	**Grand total**
No. outbreak event		14	13	18	2	3	17	67
Population	Animal outbreak	14	7	17		3	3	44
	Human outbreak	–	3	–	1	–	13	17
	A^*^ and H^*^	–	1	1	1	–	1	4
Death	Animal	14	1	9	–	–	3	27
	Human	–	4	1	–	–	11	16
Intervention	Awareness	–	1	–	1	–	3	5
	Contact tracing	–	–	1	–	–	1	1
	Isolation	–	–	1	–	–	1	1
	Animal movement control	10	4	–	–	–	3	17
	Surveillance	–	–	4	–	–	–	4
	Supportive therapy/care	–	–	1	–	–	–	1
	Animal vaccination	–	–	13	–	1	–	14

A^*^ and H^*^: Animal and Human outbreaks.

**Figure 1:**
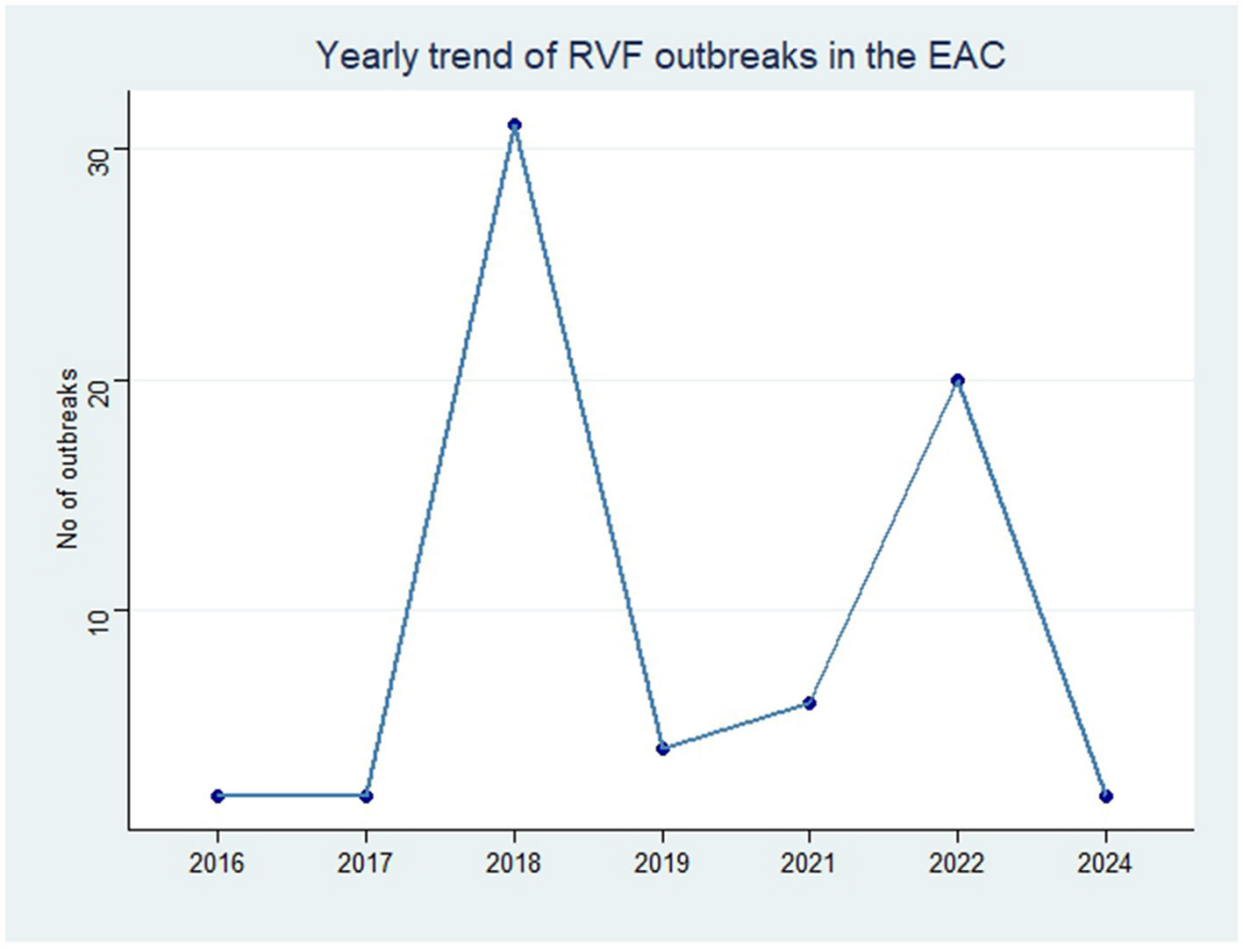
Geographical distribution of RVF outbreak events reported in EAC partner states through the Program for Monitoring Emerging Diseases (ProMed), 2010–2024. The number in parentheses represent the number of RVF outbreak events per region.

Geographical distribution of RVF outbreak events in EAC countries varied both within and between the countries (Figure 1). We first report that RVF outbreak events in Burundi were predominantly reported in the northern areas of the country. These areas included the Ngozi district, which had a high number of four RVF outbreak events. Next, in Rwanda, RVF outbreak events were largely distributed in the south and south-eastern areas, where six RVF outbreak events were reported in the Ngoma district (southeast). RVF outbreaks in northern Burundi (Ngozi district) and southeastern Rwanda (Ngoma district) are likely influenced by specific ecological and environmental factors, such as livestock density, proximity to wetlands, and agricultural practices. However, limitations in available data prevented a detailed, granular analysis of these factors.

Moreover, the RVF outbreak events in Uganda have primarily been reported throughout the southern and central areas of the country, where the district of Isingiro (south) had reported three RVF outbreak events, which exceeds outbreak numbers in other southern areas. In Kenya, RVF outbreak events have been reported across the country's western, central, northern, and eastern regions. Finally, RVF outbreak events in Tanzania were reported in north-eastern regions of the country, including the Arusha, Manyara and Tanga areas.

**Figure 2:**
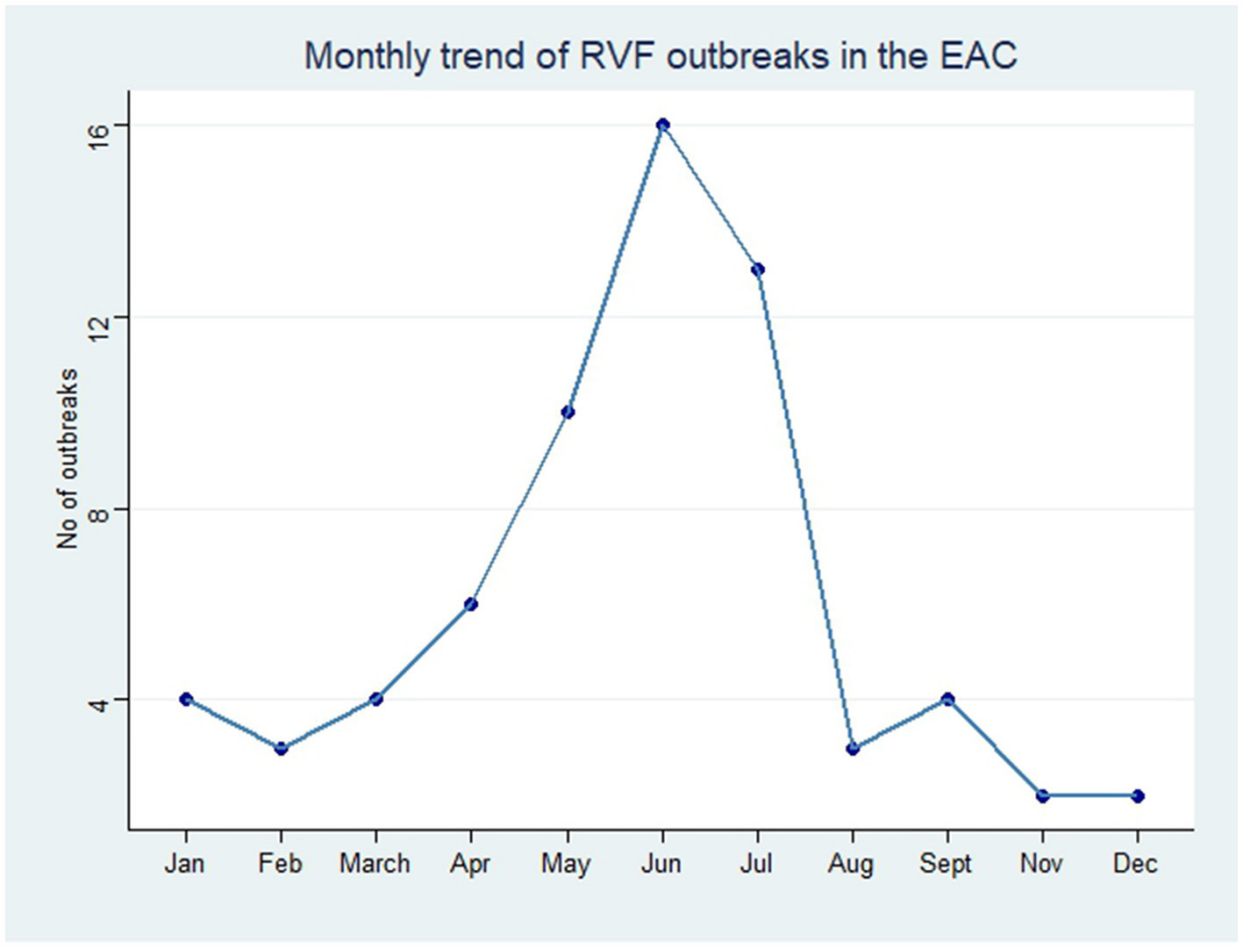
Monthly trend of RVF outbreak events reported in EAC partner states through Program for Monitoring Emerging Diseases (ProMed), 2010–2024.

**Figure 3:**
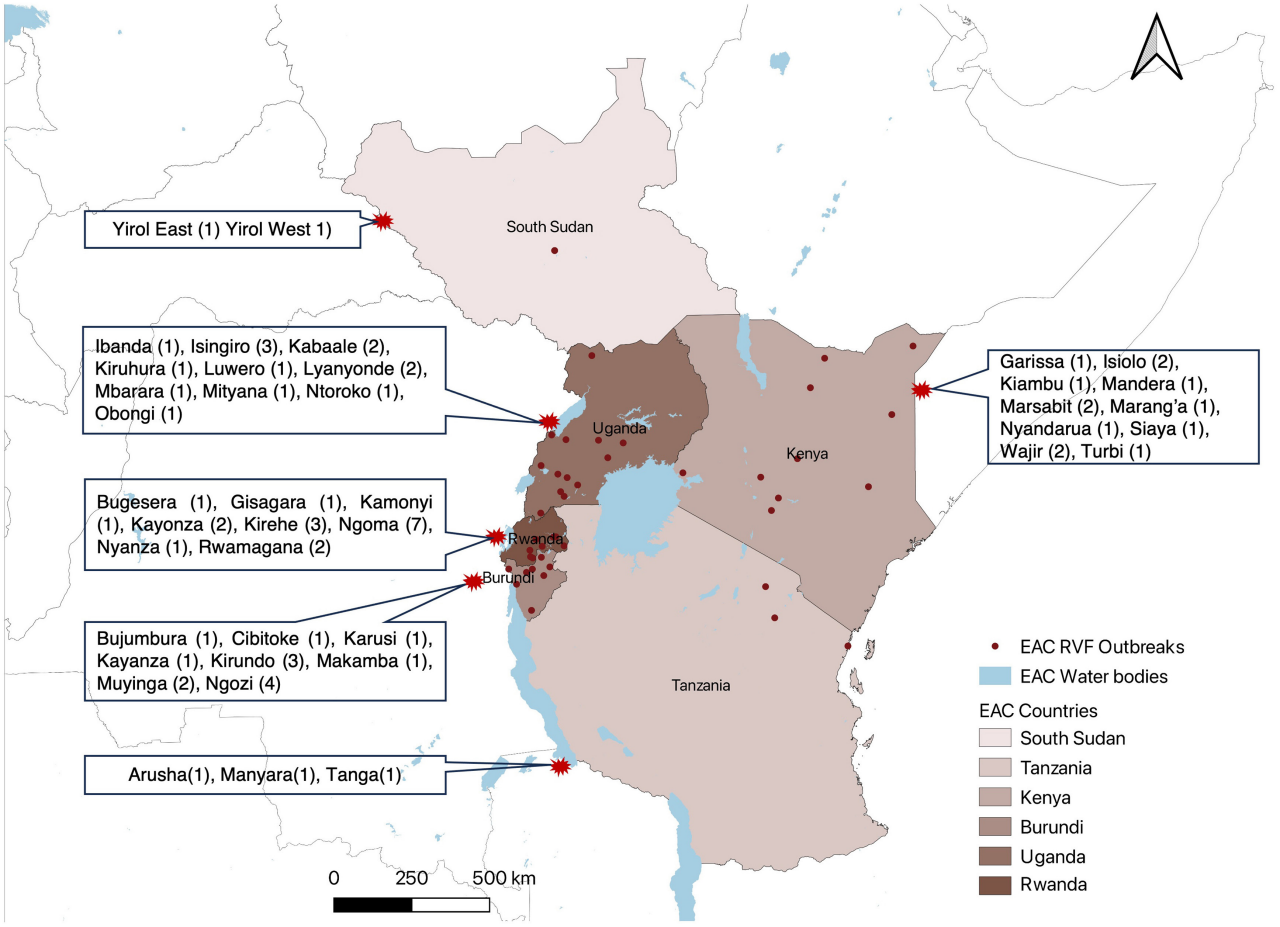
Yearly trend of RVF outbreak events reported in EAC partner states through Program for Monitoring Emerging Diseases (ProMed), 2010–2024.

In order to analyze the temporal distribution of RVF outbreak events, we quantified the year and month of occurrence of each outbreak in EAC (Figures 2, 3). The monthly trend demonstrated fluctuations in the occurrence of RVF outbreak events within the EAC partner states (excluding DRC). The number of outbreak events increased from three in the month of January to the peak with 16 RVF outbreak events in May–June. The month of August had two RVF outbreak events and the number increased to four in the month of September. The trend characterized by low occurrence of RVF outbreak events was in the month of October, November and December with the number ranging from 0 to 2 outbreak events (Figure 2).

The annual trend indicated that from 2010 to 2024 the peak of RVF outbreak events was in 2018 which recorded occurrence of 30 outbreak events, and the lowest record of zero RVF outbreak events in 2020 (Figure 3).

## Discussion

In our study, we used data from ProMed to obtain the number of RVF outbreak events in the member countries of the Eastern Africa Community from the years 2010 to 2024. From a spatial point of view, our results indicated that more than 65 RVF outbreak events were reported in EAC countries. In a temporal perspective, our review suggests that the month of June corresponds to the greatest likelihood of an outbreak event occurring. Our retrospective analysis has also highlighted the substantial increase in outbreak events in the year of 2018. We highlight the fact that the distribution of RVF outbreak events in EAC tends to be substantially higher in the months of May and July, indicating an established zoonotic cycle. The regular enzootic cycle, with limited transmission among animals, and the more severe epizootic/epidemic cycle during years/months of heavy rainfall.

The distribution of RVF outbreak events in Eastern Africa countries has been associated with specific climatic and environmental conditions. These outbreak events are reoccurring at irregular intervals following heavy rainfall with periods of flooding, which create ideal breeding conditions for the mosquito species that transmit the virus (26–28). The spread of RVF in recent years has been a major public health threat in the EAC region. Burundi had not previously documented larger RVF outbreak events. However, in 2022, an explosive RVF outbreak occurred, affecting ~13 provinces within the country. This outbreak posed a significant challenge to livestock farming and underscored the fragility of food security.^1^ It is possible that RVF outbreak events have been undetected and undocumented prior in Burundi, and this could be due to the inadequacy of surveillance systems, the country's capacity for diagnosis, and other contingency measures.^2^ However, neighboring countries including Rwanda had epidemiological evidence of experiencing larger RVF outbreak events that were reported in 2018 (20).^3^ Similarly, RVF outbreak events in Rwanda could have been under reported, as prior evidence indicated sero-prevalence in humans and animals (29). In Tanzania, RVF outbreak events have been re-occurring with an epidemics interval of 10–20 years since the 1930s (30, 31). In such endemic settings, sporadic RVF outbreak events are expected to occur, hence the 2018 RVF outbreak in Tanzania was detected promptly by the national surveillance (32). The wide spread 2018 RVF outbreak has been reported in Uganda through the National Viral Hemorrhagic Fever Surveillance System (33). The impact of the 2018 RVF outbreak has been confirmed by the Ministry of Health for Kenya, and affected humans and animals in several areas of the country (34). Similarly, this larger 2018 RVF outbreak was declared by the Ministry of Health and the Ministry of Livestock and Fisheries in South Sudan.^4^

The yearly trend of RVF outbreak events reported in EAC peaked in 2018 following the excessive rainfall caused by El Niño-Southern Oscillation (ENSO), and flooding mosquito vector habitats, similar results were reported in another study.^5^ These outbreak events usually occur during peak ENSO conditions (December–January) and subside by February to March. However, in 2018, there was an unanticipated deluge following a prolonged and widespread drought from September 2017 to February 2018. Despite early warnings of potential RVF outbreaks, the peak in May-June was unexpected, aligning with findings from other studies (see text footnote 5). The eastern part of Africa has been reported to experience a rainy season from March to May (35). Here, we speculate that the wet months from March to May augment the proliferation of RVF vectors (i.e., mosquitoes), thus contributing to a greater number of outbreak events in the months following the rainy season as indicated by Nosrat et al. (36). Additionally, extreme climatic events have been described as a catalyst for mosquito-borne diseases in an era of climate change (37, 38). The increased mosquito population, and consequently increased RVF-infected mosquitoes can cause significant outbreak events in both livestock and humans, especially for individuals in close contact with infected animals.

In addition to mosquito transmission, RVF can spread to humans through direct contact with infected livestock or through inhalation of virus particles during animal slaughter (39, 40). During RVF outbreak events, the reported mortality in human and animals was not surprising. RVF case fatality rates in humans was estimated to be high in a systematic review and meta-analysis study that pooled contemporary epidemiological data in Africa (41).

Interventions and control measures implemented during RVF outbreak events in East African countries such as the control of animal movement, animal vaccination campaigns, disease awareness, active surveillance, contact tracing, supportive therapy/care, and isolation are well known.^6^ However, the breadth and complexity of factors contributing to the risk of an RVF outbreak include climate change, the shifting distribution of vector and host species, absence of an approved human vaccine, insecticide resistance, and international travel and trade, which remain major challenges to controlling the spread of the virus (11). As climate change and extreme weather events increase in frequency, there is growing concern that RVF outbreaks will become more severe and numerous in the near future.

The limitations of this paper stem from its reliance on data gathered solely from a single event surveillance database. However, the authors justify this choice by highlighting that this event surveillance system, known as ProMed, is the largest global system for monitoring emerging diseases. Additionally, it is worth noting that ProMed employs a combination of artificial intelligence and human data moderation, enhancing its data curation capabilities. Additionally, while we recognize the importance of various environmental and socio-economic risk factors in contributing to RVF outbreaks, this study focused on the epidemiological patterns and spatial distribution of outbreaks. We plan to include a more detailed analysis of these risk factors in future studies to better understand RVF dynamics.

## Conclusion and recommendations

The recurrent and widespread outbreaks of RVF in East Africa underscore the urgent need for a coordinated, interdisciplinary approach to effectively control and prevent this disease. This approach must emphasize robust surveillance and early detection, comprehensive research and monitoring, seamless data integration, and targeted capacity building. Additionally, integrating policies, fostering community engagement, advancing vaccine and treatment development, strengthening climate resilience, enhancing education, and encouraging international collaboration are essential pillars in building a resilient and responsive global health system. Together, these efforts will empower both local and global communities to respond swiftly and effectively to RVF and other emerging health threats.

## Data Availability

Publicly available datasets were analyzed in this study. This data can be found at: https://promedmail.org/.

## Footnotes

^1^ https://reports.unocha.org/en/country/burundi/

^2^ https://www.ilri.org/news/burundi-steps-surveillance-and-response-efforts-control-rift-valley-fever

^3^ https://apps.who.int/iris/bitstream/handle/10665/273028/OEW27-300606072018.pdf

^4^ https://www.afro.who.int/news/south-sudan-declares-rift-valley-fever-outbreak-parts-eastern-lakes-state#:~:text=South%20Sudan%20declares%20Rift%20Valley%20fever%20outbreak%20in%20parts%20of%20Eastern%20Lakes%20State-12%20March%202018&text=Juba%2C%2012%20March%202018%20%E2%80%93%20The,Counties%20of%20Eastern%20Lakes%20State

^5^ https://ui.adsabs.harvard.edu/abs/2018AGUFMGH14A..07A/abstract

^6^ https://www.woah.org/en/disease/rift-valley-fever/
